# Racial discrimination and life satisfaction among Black Canadians: the mediating role of social support and the moderating roles of gender and place of birth

**DOI:** 10.3389/fpsyg.2025.1663250

**Published:** 2025-12-05

**Authors:** Jude Mary Cénat, Shruti Mistry

**Affiliations:** 1 School of Psychology, University of Ottawa, Ottawa, ON, Canada; 2 Interdisciplinary Centre for Black Health, University of Ottawa, Ottawa, ON, Canada; 3 University of Ottawa Research Chair on Black Health, Ottawa, ON, Canada

**Keywords:** racial discrimination, life satisfaction, social support, gender, place of birth, Black individuals, Canada

## Abstract

**Introduction:**

This study used an intersectional theoretical framework to investigate the association between racial discrimination experience and life satisfaction among Black individuals aged 15 to 40 years old in Canada, exploring the mediation role of social support and the moderation role of gender and place of birth.

**Methods:**

A convenience sample of 860 participants completed questionnaires assessing life satisfaction, everyday racial discrimination, social support, and sociodemographic information.

**Results:**

Findings showed that participants exposed to higher levels of everyday racial discrimination had the lowest scores of life satisfaction, *W* (3, 409) = 5.74; *p* < 0.001. Regression analyses showed that everyday racial discrimination negatively predicted life satisfaction (*β* = −0.15, *p* < 0.001), while social support (*β* = 0.38, *p* < 0.001) positively predicted it. Results from the mediation moderated model revealed a negative association between racial discrimination and life satisfaction (*β* = −0.34, *p* < 0.001), which was partially mediated by social support (*β* = 0.37, *p* < 0.001).

**Conclusions:**

This research highlights the adverse effects of racism on Black individuals’ life satisfaction and underscores the role of social support in explaining these effects. Clinicians are encouraged to adopt an intersectional approach, especially for Black women and gender-diverse Black individuals who are at a higher risk of experiencing the impact of racial discrimination on life satisfaction.

## Introduction

Racial discrimination is a process whereby prejudice, such as racial stereotypes, translate into discriminatory behaviors against a group of racialized people (Cénat, 2023; Utsey et al., 2000). This type of discrimination can occur at interpersonal, institutional, systemic, or structural levels, all of which affect an individual’s daily life (Williams et al., 2022). The prevalence of racial discrimination toward various groups of color has been well documented in countries around the world such as the United States (Lee et al., 2019; Williams and Mohammed, 2013), the United Kingdom (Hackett et al., 2020), and New Zealand (Harris et al., 2006; Houkamau et al., 2017) among others. Although colorblindness is pervasive in Canadian society, research consistently shows that Canada is no exception to racial discrimination and its harmful consequences for people of color (Banting and Thompson, 2021; Cénat et al., 2025b; Cénat et al., 2025c; Godley, 2018; James, 2010; Moshirian Farahi and Cénat, 2025; Williams et al., 2025). In a social context, colorblindness refers to the belief, attitude, or practice of claiming not to “see” race or racial differences. It is often framed as treating all people the same, regardless of skin color or ethnicity, but it contributes to reinforcing systemic racism (James, 2010). Black people in Canada are exposed to different forms of racial discrimination at different places in society including health care settings, educational institutions, and workplaces (Beiser et al., 2001; Cénat et al., 2022a; Clark et al., 2014; Currie et al., 2012; Mahabir et al., 2021). When compared to other ethnic and racial groups in Canada, Black people are among those who experience higher levels of racial discrimination (Godley, 2018).

### Impacts of racial discrimination on physical and mental health, and life satisfaction

Studies have shown that racial discrimination is associated with poorer mental health outcomes in Black, Indigenous, and People of Color (BIPOC) populations of all ages (Cénat et al., 2023b; Cénat et al., 2022b; Kogan et al., 2022; Paradies, 2006; Paradies et al., 2015; Williams et al., 2019; Williams and Williams-Morris, 2000; Yusuf et al., 2022). When compared to their White counterparts, Black people in particular developed symptoms of anxiety and depression when exposed to racial discrimination (Cénat et al., 2025a; Kogan et al., 2022; Seaton et al., 2008; Moshirian Farahi and Cénat, 2025). Studies also showed that racial discrimination has a major impact on Black people’s physical health, including elevated rates of hypertension, diabetes, and heart disease (Fincher et al., 2004; Gagné and Veenstra, 2017; Javed et al., 2022). Subtle and frequent forms of discrimination, such as microaggressions, increase the risk of Black people developing illness and disease, who are already subject to a higher risk of death and mortality from a young age (Gran-Ruaz et al., 2022; Williams and Mohammed, 2009). According to Williams et al. (2021), microaggressions are defined as subtle, often unintentional, verbal or nonverbal interactions that convey bias or discrimination toward marginalized groups. These consequences of racism on physical and mental health of Black people and other complex social and economic impacts also influence their life satisfaction (Barnes and Lightsey, 2005; Broman, 1997; Cénat, 2023). Although the association between experience of racial discrimination and life satisfaction is less explicitly explored in Canada, some research has emerged on life satisfaction within racialized communities (Foran, 2024; Government of Canada, 2022; Mata, 2002; Medina et al., 2019). Recent national surveys also show that racialized Canadians report high rates of discrimination and its negative effects on well-being (Environics Institute for Survey Research and the Canadian Race Relations Foundation, 2024; Statistics Canada, 2024, 2025). However, few studies have explicitly examined the link between racial discrimination and life satisfaction, and even fewer have focused on Black people, who are among those most likely to experience racism (Cénat et al., 2022a). This gap underscores the need for more research directly investigating how experiences of racism shape life satisfaction among Black populations in Canada. Elsewhere, a study conducted among Indigenous people in Australia showed that participants who experienced moderate to high levels of racial discrimination reported low life satisfaction (Thurber et al., 2021). Another study conducted in the United States among a racially diverse sample showed that despite the presence of two mediators (community satisfaction and friendship satisfaction), there remained a significant negative association between racial discrimination and life satisfaction (Franco, 2019).

The concept of life satisfaction refers to how someone cognitively judges all parts of their life (Diener et al., 1985; Kasprzak, 2010). Increasing experiences of perceived discrimination are associated with poorer health status, perceptions of one’s quality and conditions of life, and consequently, poorer life satisfaction (Cénat, 2023; Otiniano and Gee, 2012). Another factor that can help explain the impact of life satisfaction is stress. Individuals facing racial discrimination may be subject to experiencing higher levels of stress, whether that is at the microlevel (i.e., in everyday encounters), or at a macrolevel (i.e., in institutions and systems; Essed, 1991; Harrell, 2000). These factors also contribute to the hypervigilance and minority stress which Black people disproportionately experience in Western societies, impacting their quality of life (Carter et al., 2004). Past research has shown that all forms of race-related stress have negative implications for life satisfaction in Black American populations (Carter and Reynolds, 2011; Utsey et al., 2008). This can partly be explained by Harrell’s (2000) race-related stress framework and the complex racial trauma theory by Cénat (2023). The first is a multidimensional construct that integrates the experiences of racism between people or groups and environments that have the potential to impact their well-being. This framework helps explain how race-related stress is linked to lower life satisfaction, specifically in Black populations (Broman, 1997; Utsey et al., 2000; Utsey et al., 2008). The second stipulates that “racial trauma surrounds the victims’ life course and engenders consequences on their physical and mental health, behavior, cognition, relationships with others, self-concept, and social and economic life” (Cénat, 2023). While racism is deleterious to physical and mental health and impacts one’s overall life satisfaction, a plethora of protective factors can help mitigate the effects of racial discrimination on everyday life.

### Coping, social support, and life satisfaction

Life satisfaction extends beyond basic needs—such as stable housing, financial security, and personal health—and reflects an individual’s overall subjective well-being, encompassing both cognitive and affective evaluations of life (Diener et al., 1985). When our quality of life is threatened with adversity, we count on different forms of coping, such as active strategies (e.g., seeking information), which are used to confront the stressor, or passive strategies (e.g., avoidance), which involve ignoring the stressor (Folkman and Moskowitz, 2004). Depending on the culture and context, different coping strategies can be adaptive to different degrees (Driscoll et al., 2015). Research has shown that Black Americans who used active coping strategies experienced reduced symptoms of depression, posttraumatic stress disorder (PTSD), and race-related stress, whereas passive coping worsened existing symptoms or increased the risk of developing new mental or physical health problems (Mekawi et al., 2022; West et al., 2010). Other researchers found that Black Americans were less likely to engage in problem and emotion-focused coping when facing race-related stressors compared to generic life stressors and hassles (Joseph and Kuo, 2009).

In most contexts, supportive relationships are vital to well-being and are one of the strongest determinants of life satisfaction (Kasprzak, 2010). Social support can be characterized as having a readily available network of family, friends, neighbors, and community members to provide psychological, physical, or financial help (Cénat et al., 2021; Driscoll et al., 2015; Kasprzak, 2010). According to Kasprzak (2010), a dependable and accepting support network is essential to maintaining life satisfaction. In Black communities, collective efficacy and sociocultural resources are used as a common coping mechanism to protect against racial discrimination (Driscoll et al., 2015; Utsey et al., 2006). A systematic review on how Black people cope with racism revealed that one of the most common forms of resources that Black Americans used to cope with pain, either physical or from racism, was social support (Jacob et al., 2022). Interestingly, social support may be more effective for coping with certain types of racism more than others. Driscoll et al. (2015) found that collective efficacy and problem-focused coping were effective in reducing impacts of cultural discrimination, referring to the representations of racial or ethnic groups as inferior to others, but not institutional race-related stress. Similarly, avoidance was more helpful for coping with interpersonal discrimination (Utsey et al., 2000).

### Gender and generational differences related to discrimination and coping strategies

Gender identity can be defined as one’s personal conception of their gender under a social, cultural, and behavioral context (Lev, 2013). Certainly, gender identity impacts our socialization, and consequently, how people cope with stressors in their everyday lives (Brownlow et al., 2019). In Black communities, where racial discrimination disproportionately impacts levels of stress, men often turned to more internalizing responses, acceptance, self-reliance, and developed vigilance (Lewis et al., 2012; Sullivan et al., 2021). Black women relied on talking with others, engaging in spiritual practices, seeking social support, and using emotion-focused coping strategies (Jacob et al., 2021). A systematic review of Canadian literature emphasized how Lesbian, Gay, Bisexual, Transgender, and Queer/Questioning (LGBTQ+) people of color in Canada experienced a heightened level of microaggressions, facing a double discrimination in all aspects of their lives related to their racial background and sexual orientation or gender identity (Sadika et al., 2020). Seeking social supports and reconceptualizing their identities were a few significant, protective measures that were extracted from their review. While there are few studies that have explored the coping mechanisms of Black gender minorities, social support seems to reign as an important coping strategy for these populations, regardless of race (Pflum et al., 2015). While there may be significant overlap in the strategies employed in different Black communities to protect against racism (Jacob et al., 2022), it is important to consider other aspects of identity other than race and gender, and how these may impact the coping process.

Alongside gender, place of birth is another sociodemographic characteristic that impacts coping mechanisms. Those born abroad (i.e., immigrants and refugees) are at a higher risk of experiencing racial discrimination due to language barriers, cultural differences, and being in settings that contribute to this elevated risk [i.e., schools, labor-market, housing, etc.; Banerjee (2008) and Oxman-Martinez et al. (2012)]. Indeed, facing discrimination amplifies race-related stress due to immigrant status, more so than their native counterparts (Szaflarski and Bauldry, 2019). Resilience, problem-focused coping, and social support are some examples of common coping mechanisms for racial discrimination that are seen in Korean and Chinese American immigrants (Choi et al., 2020; Guo et al., 2022; Mossakowski and Zhang, 2014). A study on African migrants in Australia found that having networks, family support, and engaging in faith-based practices helped to cope with racial and resettlement stress (Ikafa et al., 2021). Along with racial discrimination, immigrants facing minority and immigrant-related stressors faced barriers in developing coping strategies and generating social capital (Valentín-Cortés et al., 2020). While social capital has shown to play a crucial protective role to racism (Husbands et al., 2022), the pervasive nature of systemic and interpersonal racism has shown to disrupt the development of social capital, and thereby well-being (Brondolo et al., 2012). Controversially, racial discrimination has also shown to have a greater impact on Black individuals born in Canada from second generations in Western countries (Cotter, 2022). Studies explained this observation by the role of racial identity and the complex racial trauma experienced by Black individuals born and raised in a minority context where racism is pervasive since childhood (Cénat, 2023; Sellers and Shelton, 2003). To date, no research has yet examined how gender and place of birth influence life satisfaction among Black Canadians who experience racial discrimination. However, understanding how gender and place of birth shape coping mechanisms is essential, as these factors directly influence individuals’ overall life satisfaction. Coping strategies that foster resilience and social connectedness can mitigate the negative psychological impact of discrimination, thereby enhancing well-being. Thus, exploring these dynamics offers crucial insight into how the experience of racial discrimination translates into disparities in life satisfaction among Black Canadians.

### Theoretical framework: intersectionality

People are simultaneously exposed to different kinds of discrimination related to their skin color, gender, ethnicity, sexual orientation, disability, and more. Hence, it is important to consider the multiple systems of oppression that racialized people are subject to. Coined by Kimberlé Crenshaw (1989), the term intersectionality highlights the multidimensional experiences of discrimination and oppression that arise through possessing multiple marginalized identities. It also “examines how distinctive social power relations mutually construct each other” (Bowleg, 2008, p. 313). As such, experiences of racial discrimination are not only a product of one’s race. Several studies support that Black non-binary people experience discrimination related to both their skin color and their gender identity, while cisgender Black men and women, and White non-binary people may face different experiences of discrimination related to one aspect of their identity (Biello and Hughto, 2021; Clark et al., 2023). In addition, recent research showed that the social support received by Black non-binary individuals may be different than that received by Black cisgender individuals and White non-binary individuals, such as facilitating their coming out, gender transition, social adjustment, and self-acceptance (Graham et al., 2014; Quinn et al., 2023). In a study conducted by Jones et al. (2022), they found that a higher frequency of gendered racism reduced the level of perceived social support and increased depressive symptoms. This finding reveals how the layer of a marginalized gender identity influences the level of engagement with support networks and preferences for other coping mechanisms. Still, there is a dearth of research on the well-being of Black non-binary and other gender-diverse groups. Building on the intersectionality framework, life satisfaction can be understood as shaped by the cumulative effects of overlapping systems of oppression and privilege. Individuals holding multiple marginalized identities, such as being both Black and gender-diverse, may experience compounded discrimination that limits access to social support and resources essential for well-being, thereby reducing overall life satisfaction.

### The present study

**Figure 1:**
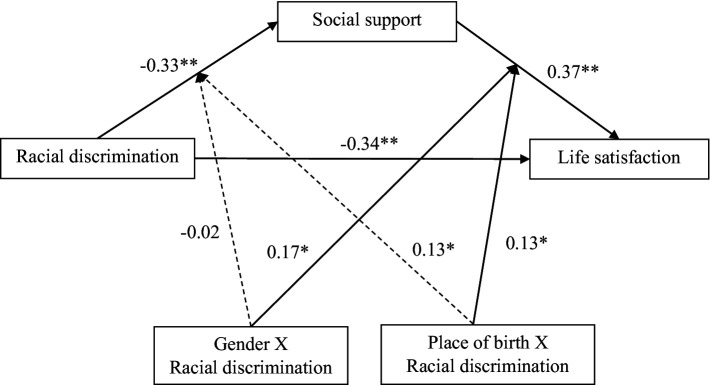
Conceptual moderated mediation model of racial discrimination, social support, life satisfaction, gender, and place of birth.

While many studies have investigated the protective role of social support in the association between racial discrimination and mental health (Brownlow et al., 2019; Cénat et al., 2021; Cénat et al., 2024b; Driscoll et al., 2015; Joseph and Kuo, 2009; Liao et al., 2016; Prelow et al., 2006; Seaton et al., 2008; Utsey et al., 2006), none address the underlying mechanisms that affect life satisfaction, according to an intersectional framework in the Canadian context. Given the observed gaps and the empirical observations made in the relationship between racial discrimination, social support, life satisfaction, and gender, using data from the Black Community Mental Health (BCoMHeal) project (Cénat et al., 2023a), the current study aims to (a) examine the life satisfaction and racial discrimination of Black individuals in Canada according to various sociodemographic characteristics; (b) examine the association between racial discrimination and life satisfaction among Black individuals in Canada; (c) investigate the mediating role of social support in the association between racial discrimination and life satisfaction of Black individuals in Canada; and (d) examine the moderating role of gender and place of birth in the relationship between racial discrimination and life satisfaction. We hypothesize that (1) the experience of everyday racial discrimination will be associated with a lower life satisfaction; (2) among participants who experience racial discrimination, social support may explain a better life satisfaction; and (3) gender (being a woman or a gender minority) and place of birth (born outside of Canada) will negatively moderate the association between racial discrimination and life satisfaction. The conceptual model is presented in Figure 1.

## Methods

### Procedures and participants

The BCoMHeal Project (Black Communities Mental Health) is conducted by the Vulnerability, Trauma, Resilience, and Culture Laboratory (V-TRaC Lab) and the Interdisciplinary Centre for Black Health at the University of Ottawa. It aims to reduce mental health disparities among Black communities in Canada. It pursues three main goals: (1) documenting the prevalence and determinants of mental health issues among Black populations in Canada; (2) educating, raising awareness, and mobilizing Black communities around mental health; and (3) developing, implementing, and evaluating culturally adapted assessment and intervention tools in both clinical and community settings to strengthen anti-racist, evidence-based mental health care. The quantitative phase of the BCoMHeal project recruited participants through four mediums. First, posters were advertised in universities and places of worship. Second, a virtual advertisement of the study was shared through social media sites (i.e., Facebook, Twitter, Instagram) run by the Principal Investigator’s research laboratory. Third, community organizations working with Black youth sent emails to invite their peers to share the study. Fourth, through the Integrated System of Participation in Research (ISPR), undergraduate psychology students had access to participate in the survey at the University of Ottawa. All participants completed the questionnaire through the Qualtrics™ platform (Provo, USA) and informed consent was obtained prior to completing the survey. The participants in the fourth medium received a credit for their class, whereas all other participants were compensated with a $15 gift card for their participation. The University of Ottawa Research Ethics Board and the Université du Québec en Outaouais Research Ethics Board approved the research protocol. Inclusion criteria for participants consisted of (1) self-identifying as Black, (2) residing in Canada, (3) being between 15 and 40 years of age, and (4) having the ability to understand written French or English. More details on the procedures can be found in a previous publication (Cénat et al., 2021; Darius et al., 2024).

A total of 860 participants (*M*_age_ = 25.0 years, *SD* = 6.3), predominantly born in Canada (79.1%), and women (75.6%) completed the survey. The sociodemographic characteristics of the participants are summarized in Table 1.

**Table 1:** Prevalence of life satisfaction by sociodemographic characteristics and perceived racial discrimination among Black individuals in Canada (*N* = 860).

		Life satisfaction		Everyday racial discrimination	
Sociodemographic characteristics	*N*	*M*	*SD*	*M*	*SD*
Total	860	20.77	4.26	18.46	5.97
Age	*t*(856) = 1.98; *p* = 0.048			*t*(858) = −11.45; *p* < 0.001	
15–24 years	557	20.95	4.94	16.85	5.63
25–40 years	303	20.44	2.56	21.40	5.44
Gender	*W*(2,83) = 0.01; *p* = 0.986			*W*(2,81) = 16.23; *p* < 0.001	
Men	181	20.73	5.04	16.99	5.09
Women	650	20.78	4.09	18.98	6.19
Non-binary and other gender-diverse groups	29	20.72	2.19	15.90	3.55
Education	*W*(4,162) = 0.75; *p* = 0.562			*F*(4,855) = 46.47; *p* < 0.001	
None	45	21.67	6.13	15.29	5.24
High school	125	20.89	5.94	15.98	5.53
Incomplete postsecondary	482	20.58	3.18	20.63	5.58
Postsecondary	144	20.70	4.39	16.38	5.00
University	64	21.44	5.38	13.84	5.04
Place of birth	*t*(678) = 0.18; *p* = 0.855			*t*(856) = −8.15; *p* < 0.001	
Born in Canada	373	20.80	4.82	16.68	5.03
Born abroad	487	20.74	3.74	19.82	6.28
Household income	*W*(6,117) = 0.74; *p* = 0.619			*W*(6,124) = 12.55; *p* < 0.001	
Under $19,999	32	20.00	4.37	16.66	5.01
$20,000–$29,999	28	21.89	6.51	17.32	5.35
$30,000–$39,999	41	21.02	5.01	17.78	4.38
$40,000–$49,999	40	19.85	5.55	16.88	5.38
$50,000–$74,999	93	20.98	4.67	16.34	4.68
$75,000–$99,999	560	20.69	3.80	19.54	6.22
Over $100,000	66	21.39	4.69	14.98	4.72
Marital status	*W*(3,101) = 4.34; *p* = 0 0.006			*W*(3,106) = 143.13; *p* < 0.001	
Single	444	21.18	5.44	15.61	5.25
Married	333	20.38	2.10	22.71	4.61
Separated	51	19.55	3.37	17.84	3.61
Other	32	21.03	2.76	14.66	4.50
Employment status	*t*(502) = −1.12; *p* = 0.265			*t*(751) = 7.74; *p* < 0.001	
Yes	567	20.64	3.96	19.46	6.30
No	293	21.00	4.79	16.51	4.71
Religious involvement	*W*(4,229) = 1.09; *p* = 0.364			*W*(4,260) = 89.89; *p* < 0.001	
Never	89	20.97	4.74	15.45	4.34
Rarely (once a year)	112	20.67	5.23	15.71	5.03
Sometimes (at least 4 times a year)	439	20.56	2.89	21.56	4.92
Often (at least once a month)	124	20.71	5.48	16.09	5.57
Very often (2–3 times a week)	96	21.74	5.70	13.33	5.29
Everyday racial discrimination		*W*(3, 409) = 5.74; *p* < 0.001			
Rarely	201	22.01	5.40		
Sometimes	197	20.61	4.19		
Often	211	20.15	4.76		
Very often	251	20.41	2.12		

*t*, independent *t*-test.

*W*, Welch’s test.

*F*, One-way ANOVA.

### Measures

#### Sociodemographic characteristics

Participants completed a sociodemographic questionnaire that collected information on gender, age, education level, marital status, employment status, place of birth, household income, and level of participation in religious ceremonies. The sociodemographic characteristics of the sample are presented in Table 1.

#### Everyday discrimination scale

Discriminatory racial experiences were evaluated using the Everyday Discrimination Scale (EDS; Williams et al., 1997). The EDS measures the perceived source of racial discrimination and the frequency of lifetime daily discrimination. An example of a question includes: “*People act as if they think you are not smart*.” Items are rated on a 6-point scale: Almost everyday (6); At least once a week (5); A few times a month (4); A few times a year (3); Less than once a year (2); Never (1). A higher total score signifies a higher frequency of perceived discrimination by the individual. The EDS was developed and widely used among Black communities in the United States with good internal consistency (usually Cronbach’s *α* was more than 0.85; Gonzales et al., 2016). Cronbach’s alpha in our sample was 0.90.

#### Life satisfaction

The Satisfaction with Life Scale (SWL) is a 5-item self-report questionnaire (Diener et al., 1985). The scale evaluates one’s subjective, global cognitive judgments of their satisfaction with life by rating 5 items on a 7-point Likert scale. Sample items include: “*In most ways, my life is close to my ideal,*” and “*If I could live my life over, I would change almost nothing*.” Items are rated from (1) Strongly Disagree to (7) Strongly Agree. This scale shows good convergent validity with similar scales that measure subjective well-being. A higher score signifies a higher level of satisfaction with life. Cronbach’s alpha in our sample was 0.87.

#### Multidimensional scale of perceived social support

The Multidimensional Scale of Perceived Social Support is a 12-item self-report questionnaire (Zimet et al., 1988). It evaluates sources of social support, which tend to group into three factors: family (Items 3, 4, 8, and 11); friends (Items 6, 7, 9, and 12); and significant other (Items 1, 2, 5, and 10). Sample items include: *“There is a special person who is around when I am in need,”* and *“I can count on my friends when things go wrong.”* Items are rated on a 7-point scale: (1) Very Strongly Disagree to (7) Very Strongly Agree. Cronbach’s alpha coefficients for significant other, family, and friends’ subscales were 0.91, 0.87, and 0.85, respectively (total reliability score of 0.88; Zimet et al., 1988). It is widely used among Black individuals in minority contexts with excellent internal consistency (e.g., Cronbach’s *α* = 0.93; Webb Hooper et al., 2013). Cronbach’s alpha in our sample was 0.90.

### Statistical analyses

We used the Statistical Package for the Social Sciences (SPSS) Version 30 for descriptive and mean comparison analyses and STATA/SE (version 19.5) for regression and mediation-moderation model (Cain, 2021). Missing data was addressed using multiple imputation, generating seven imputed datasets. The first dataset was used for all analyses. We computed descriptive analyses to examine the mean differences according to sociodemographic characteristics. We compared mean scores of life satisfaction according to sociodemographic characteristics, using independent samples *t*-tests and one-way analysis of variance (ANOVA). When the assumption of homogeneity of variances was violated, results from t-tests with unequal variances assumed and Welch’s ANOVA were reported. We also performed post-hoc analyses on significant results of the ANOVA *F*-tests using Tukey’s Honest Significant Difference (HSD) test. Given the importance of the variable of everyday racial discrimination, it was categorized into four groups based on quartiles: first quartile (0–25th percentile), second quartile (26–50th percentile), third quartile (51–75th percentile), and fourth quartile (76–100th percentile). This procedure, previously used in studies among racialized populations in Canada (Cénat et al., 2021; Moshirian Farahi and Cénat, 2025), allowed for the examination of differences across various levels of discrimination. The categorical variable was used only in proportion analyses in order to observe differences between levels of discrimination. A two-step multiple linear regression analysis was conducted to test the association between everyday racial discrimination (Model 1) and social support (Model 2) with life satisfaction, after controlling for sociodemographic factors. The assumption of multicollinearity was assessed using Variance Inflation Factor (VIF) values. In the regression model, categorical variables included: gender, age, marital status, place of birth, education, income, and employment status. The continuous variables included everyday discrimination, social support, and religious participation. Standardized coefficients, adjusted R^2^, and F-tests were also reported. Considering the results of the multiple linear regression models, structural equation modeling (SEM) was used to estimate the direct and indirect effects of racial discrimination on life satisfaction through social support. The mediator for this model was social support and the moderators were gender (men, women, and non-binary) and place of birth (born in Canada and born abroad). We used the Full Information Maximum Likelihood (FIML) for the estimation of the parameter and model fit. We assessed the fit of the SEM using the root mean square error of approximation (RMSEA; value less than 0.06); Tucker-Lewis Index (TLI); and Comparative Fix Index (CFI; value greater than 0.9 indicates a good fit; Hu and Bentler, 1999).

## Results

### Life satisfaction

Results showed significant results in mean life satisfaction among levels of perceived racial discrimination, *W* (3, 409) = 5.74; *p* < 0.001. *Post hoc* results showed that participants who rarely perceived racial discrimination (*M* = 22.01, *SD* = 5.40) had higher levels of life satisfaction compared to those who perceived racial discrimination sometimes (*M* = 20.61, *SD* = 4.19), often (*M* = 20.15, *SD* = 4.76), and very often (*M* = 20.41, *SD* = 2.12). Participants aged between 25 and 40 years old experienced lower life satisfaction (*M* = 20.44, *SD* = 2.56) compared to those aged 15 to 24 years old (*M* = 20.95, *SD* = 4.94), *t* (856) = 1.98, *p* = 0.048. Results also showed significant differences in mean score life satisfaction among marital status, *W* (3, 101) = 4.34, *p* = 0.006. Separated (*M* = 19.55, *SD* = 3.37) and married participants (*M* = 20.38, *SD* = 2.10) had significantly lower life satisfaction scores than single participants (*M* = 21.18, *SD* = 5.44). There were no significant differences observed for gender, education, place of birth, household income, employment, or participation in religious activity for overall life satisfaction. All the results are presented in Table 1.

### Perceived racial discrimination

Participants aged between 25 and 40 years old were exposed to higher levels of racial discrimination (*M* = 21.40, *SD* = 5.44) compared to those aged 15 to 24 years old (*M* = 16.85, *SD* = 5.63). Regarding gender, results showed a significant group difference in mean score of racial discrimination among genders, *W* (2, 81) = 16.23, *p* < 0.001. Post hoc results showed that women had higher mean scores of racial discrimination (*M* = 18.98, *SD* = 6.19) than men (*M* = 16.99, *SD* = 5.09), and non-binary and other gender-diverse groups (*M* = 15.90, *SD* = 3.55). Group differences were also observed for education, *F* (4, 855) = 46.47, *p* < 0.001. Post hoc results showed that participants without a post-secondary certificate or diploma that attended a post-secondary institution experienced higher levels of racial discrimination (*M* = 20.63, *SD* = 5.58) than participants who had received no education (*M* = 15.29, *SD* = 5.24), had a diploma or high school equivalency (*M* = 15.98, *SD* = 5.53), a postsecondary certificate or diploma below a bachelor’s degree (*M* = 16.38, *SD* = 5.00), and a bachelor’s degree or above (*M* = 13.84, *SD* = 5.04). Regarding place of birth, participants who were born abroad reported higher levels of racial discrimination (*M* = 19.82, *SD* = 6.28) than those born in Canada (*M* = 16.68, *SD* = 5.03), *t* (856) = −8.15, *p* < 0.001. Regarding income, results showed a significant difference in mean scores for racial discrimination according to income, *W* (6, 124) = 12.55, *p* < 0.001. Post hoc results showed that participants who have a household income of $75,000 to $99,999 experienced higher levels of racial discrimination (*M* = 19.54, *SD* = 6.22) compared to those with an income of $50,000 to $74,999 (*M* = 16.34, *SD* = 4.68) and $100,000 or above (*M* = 14.98, *SD* = 4.72). Results showed significant differences in mean life satisfaction among marital status, *W* (3, 106) = 143.13, *p* < 0.001. Post hoc results showed that married participants had the highest mean score of racial discrimination (*M* = 22.71 *SD* = 4.61) than participants who were single (*M* = 15.61, *SD* = 5.25), separated (*M* = 17.84, *SD* = 3.61), and other (*M* = 14.66, *SD* = 4.50). Regarding religious involvement, results showed a significant group difference in racial discrimination between levels of religious involvement, *W* (4, 260) = 89.89, *p* < 0.001. Post hoc comparisons indicated that mean exposure of racial discrimination was higher in participants who are sometimes involved in religious activities (*M* = 21.56, *SD* = 4.92) compared to those who never participate (*M* = 15.45, *SD* = 4.34), rarely participate (*M* = 15.71, *SD* = 5.03), participate often (*M* = 16.09, *SD* = 5.57), and participate very often (*M* = 13.33, *SD* = 5.29).

### Multiple linear regression models

Two-step multiple linear regression analyses were conducted to investigate the association between everyday racial discrimination (Model 1) and social support (Model 2) after controlling for sociodemographic factors. As presented in Table 2, for the first model, everyday racial discrimination was negatively associated with life satisfaction (*β* = −0.5, *p* < 0.001). Model 1 explained only 2% of the variance. In Model 2, when social support was integrated, the association between everyday racial discrimination and life satisfaction became non-significant (*β* = −0.07, *p* = 0.100). Social support was positively associated with life satisfaction (*β* = 0.54, *p* < 0.001). Model 2 explained 15% of the variance, indicating the key role of social support in predicting life satisfaction.

**Table 2:** Multiple linear regression of factors related to life satisfaction.

	Model 1			Model 2		
	*F* (20, 839) = 1.88, *p* = 0.011, *R*^2^ = 0.02			*F* (21, 838) = 8.20, *p* < 0.001, *R*^2^ = 0.15		
Variables	Standardized *β*	*t*	*p* value	Standardized *β*	*t*	*p* value
Gender (ref: men)						
Women	0.02	0.55	0.584	0.04	1.13	0.259
Non-binary	0.03	0.79	0.428	0.06	1.41	0.159
Place of birth (ref: born in Canada)						
Born abroad	0.05	1.08	0.281	0.02	0.54	0.587
Everyday racial discrimination	−0.15	−3.47	0.001	−0.07	−1.65	0.100
Marital status (ref: single)						
Married	−0.04	−0.63	0.528	−0.03	−0.49	0.626
Separated	−0.11	−2.73	0.007	−0.09	−2.35	0.019
Other	−0.04	−0.99	0.324	−0.03	−0.69	0.491
Religious Participation	0.01	0.16	0.872	−0.04	−1.17	0.242
Age (ref: 15–24 years)						
25–40 years	−0.01	−0.12	0.905	0.00	0.00	0.997
Education (ref: none)						
High school	−0.05	−0.88	0.380	−0.04	−0.77	0.444
Incomplete postsecondary	−0.03	−0.33	0.742	−0.05	−0.59	0.553
Postsecondary	−0.05	−0.79	0.427	−0.04	−0.72	0.471
University	−0.02	−0.35	0.730	−0.05	−0.98	0.325
Income (ref: under $19,999)						
$20,000–$29,999	0.08	1.71	0.088	0.08	1.84	0.066
$30,000–$39,999	0.05	1.02	0.307	0.05	1.16	0.246
$40,000–$49,999	−0.02	−0.30	0.762	−0.02	−0.38	0.707
$50,000–$74,999	0.05	0.85	0.396	0.08	1.27	0.204
$75,000–$99,999	0.08	0.86	0.392	0.10	1.18	0.238
Over $100,000	0.08	1.31	0.191	0.08	1.54	0.123
Employment (ref: yes)						
No	0.04	1.02	0.308	0.04	1.09	0.277
Social support				0.38	11.36	<0.001

### Moderated mediation of life satisfaction

**Figure 2:**
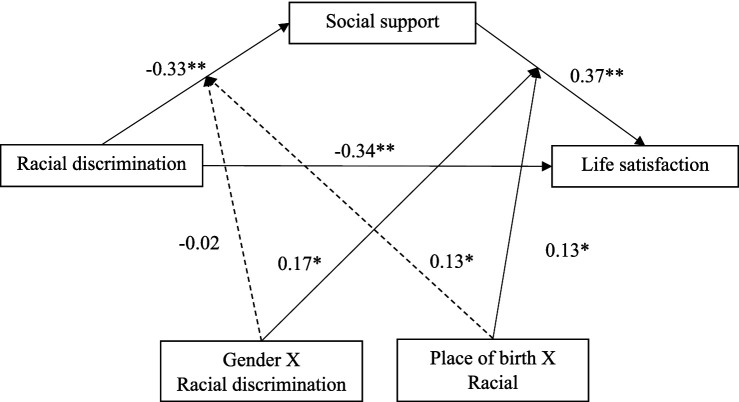
Statistical moderated mediation model of racial discrimination, social support, life satisfaction, and place of birth. Coefficient, standardized regression coefficient. CI, confidence interval. Dotted lines represent an insignificant path from the model (*p* > 0.05) whereas full lines represent a significant path (*p < 0.05; ** *p* < 0.001).

The fit for this model was excellent (CFI = 0.996; TLI = 0.951; and RMSEA = 0.030). The model showed that everyday racial discrimination was associated with lower life satisfaction (*β* = −0.34, *p* < 0.001). The association between racial discrimination and life satisfaction was positively and partially mediated by social support (*β* = 0.37, *p* < 0.001). The interaction between gender and racial discrimination was positively associated with life satisfaction (*β* = 0.17, *p* = 0.043), suggesting a moderating effect of gender in the association between racial discrimination and life satisfaction. A significant interaction between place of birth and everyday racial discrimination on life satisfaction was found (*β* = 0.13, *p* = 0.029). Results are presented in Table 3. The statistical model is presented in Figure 2.

**Table 3:** Standardized coefficients of the moderated mediation model of racial discrimination, social support, life satisfaction, gender, and place of birth.

Social support	Coefficient	*SE*	*z*	*p* value	95% CI
Gender	−0.06	0.04	−1.55	0.121	[−0.14, 0.02]
Place of birth	0.10	0.04	2.48	0.013	[0.02, 0.18]
Everyday racial discrimination	−0.33	0.09	−3.75	<0.001	[−0.50, −0.16]
Gender x Everyday racial discrimination	−0.02	0.09	−0.18	0.854	[−0.20, 0.16]
Place of birth x Everyday racial discrimination	0.13	0.06	2.02	0.043	[0.00, 0.26]

Life satisfaction	Coefficient	*SE*	z	*p* value	95% CI
Age	−0.04	0.04	−1.13	0.260	[−0.12, 0.03]
Gender	0.04	0.04	1.19	0.235	[−0.03, 0.12]
Place of birth	0.02	0.04	0.46	0.646	[−0.06, 0.10]
Everyday racial discrimination	−0.34	0.08	−4.02	<0.001	[−0.50, −0.17]
Gender x Everyday racial discrimination	0.17	0.09	2.02	0.043	[0.01, 0.34]
Place of birth x everyday Racial discrimination	0.13	0.06	2.18	0.029	[0.01, 0.25]
Social support	0.37	0.03	12.34	<0.001	[0.31, 0.43]

Coefficient, standardized coefficient; CI, confidence interval.

## Discussion

The first objective of this study was to explore the level of life satisfaction among Black people in Canada according to sociodemographic characteristics. First, in our sample, the mean life satisfaction score was 20.77. Hopper et al. (2022), using baseline data from the Canadian Longitudinal Study on Aging (over 50,000 adults), reported a mean score of 28.0. This comparison indicates lower life satisfaction among Black Canadians in our study relative to the general Canadian population. In addition, contrary to what was anticipated, most sociodemographic characteristics of the sample showed no significant differences, including gender, education, place of birth, household income, employment status, or religious participation. While these factors may impact life satisfaction in interaction with each other, this finding indicates that these sociodemographic characteristics alone do not have significant impacts on life satisfaction in our sample of Black Canadians. This finding could also be attributed to the fact that life satisfaction broadly measures several determinants of life conditions, and it is therefore less likely that a single characteristic could have a large enough magnitude to affect one’s overall score on life satisfaction. As such, findings only showed significant differences for marital status and age group. Those between the ages of 25 and 40 years old experienced lower levels of life satisfaction. This finding is consistent with the U-shaped happiness-age trajectory which asserts that a well-being score is higher in young age before it dips in middle age, then rises again in older age, which is found to be consistent in studies with African American populations (Blanchflower and Oswald, 2008; Zhang et al., 2017). It is important to note that this trajectory is a broad generalization and could be impacted by generational events and other sociodemographic or environmental factors. For marital status, those who were married or separated experienced lower levels of life satisfaction compared to those who were single. This is consistent with existing research that demonstrates how separated Black American women have lower life satisfaction and global happiness compared to married Black couples (Ball, 1983). One factor that may explain this difference is the socioeconomic disadvantage faced by Black families. Studies report how Black women choose not to marry due to economic instability, and Black men continue to face lower employability and educational achievement (Ball and Robbins, 1986; Hurt et al., 2014). A combination of these factors may help explain the difference in life satisfaction seen between separated and married Black couples.

As part of our moderated mediation model, we examined the association between racial discrimination and life satisfaction among Black Canadians. We hypothesized that exposure to higher levels of racial discrimination would decrease life satisfaction. The results support this hypothesis. First, the results demonstrate that those with higher levels of racial discrimination experience low levels of life satisfaction (Table 1). Second, the results of the mediated moderation model show that a strong negative association between racial discrimination and life satisfaction remains despite the moderating role of gender and the mediating role of social support. Studies conducted in the United States also report that African Americans experiencing racial discrimination and race-related stress experience negative impacts on their life satisfaction and overall well-being due to poor racial identity (Broman, 1997; Driscoll et al., 2015; Utsey et al., 2000).

Based on the current literature regarding strategies in Black communities to cope with experiences of racial discrimination, we hypothesized that social support would positively mediate the association between racial discrimination and life satisfaction. Results showed that social support positively and partially mediated this association. Past studies report similar results with improvement in physical and mental health, alongside overall well-being in other racialized populations (Hashemi et al., 2019; Jasinskaja-Lahti et al., 2006; Park and Joshanloo, 2022; Utsey et al., 2006) and Black populations (Heard et al., 2011; Mosher et al., 2006). A study that examined the impacts of social support on African American women experiencing discrimination found that social support helped to explain low depressive symptoms after their victimization (Seawell et al., 2014). In gender diverse populations, community participation and connectedness were reported to facilitate psychological well-being (Sherman et al., 2020).

This research supports existing literature suggesting that social support can buffer the negative effects of racial discrimination on mental health and overall life satisfaction, particularly among Black populations. Receiving support from friends, family, acquaintances, and even mental health professionals, whether general or tailored to the experience of discrimination, may help increase one’s life satisfaction. Clinicians have a role in helping their clients find ways to expand their social networks and connect with communities who share similar racial or ethnic identities. We must also recognize the other stressors that may be hindering their satisfaction with life, such as physical health problems, or other forms of discrimination.

We also hypothesized that gender would moderate the association between racial discrimination and life satisfaction. Indeed, the results revealed that being a woman or a gender minority exacerbates the impact that racial discrimination has on life satisfaction. This finding reveals how Black people who do not identify as male not only suffer the consequences resulting from racism, but also from sexism, transphobia, and the patriarchy. Black women and gender minorities in Canada are more likely to experience racism (Cénat et al., 2022a, 2024a; Husbands et al., 2022). This association is linked to Crenshaw’s theory of intersectionality, describing how Black women and gender minorities face discrimination in multiple facets of their identities and lives (Crenshaw, 1989). When considering Black gender minorities, Black transgender women experience disproportionate violence compared to Black men and women, as well as higher rates of polyvictimization (Sherman et al., 2022). This important finding emphasizes the need to find specific interventions for gender minority people of color and Black women. One study discovered that Black women who have a higher sense of belongingness, public regard, and overall racial identity are more likely to have better life satisfaction (Yap et al., 2011). As we know, racial identity is a protective factor for racial discrimination (Carter and Reynolds, 2011; Cotter, 2022; Sellers and Shelton, 2003). However, Black transgender men and women experience transphobia in both of their own Black and gender communities (Lacombe-Duncan et al., 2022; McCormick and Barthelemy, 2021; Rood et al., 2017). The intersection of these multiple forms of oppression can benefit from social support. Social support is one factor that explains life satisfaction among Black non-binary and other gender-diverse groups. It is also one way that is recommended to build a healthy support system. Finding a mentor, life coach, or mental health professional that identifies with one’s marginalized identities can significantly impact the experience and help-seeking behavior of the client; however, it may not always be feasible depending on the supports available. Since community connection for Black non-binary and other gender-diverse people has been found to improve help-seeking behaviors and overall mental health (Sherman et al., 2022), social support must be leveraged and studied longitudinally to see if it can be a strong coping mechanism for life satisfaction across the lifespan.

### Limitations

Despite these important findings, there are a few limitations that must be addressed in the scope of this study. Firstly, the methodology of the present study used a cross-sectional design, which prevents us from making any causal inferences regarding the results. Longitudinal data is necessary to understand the magnitude of social support over a lifetime. Another limitation within the questionnaire is regarding gender, where the options for the self-identifying gender question included “men,” “women,” “non-binary”, and “none of these options apply to me.” Without options for transgender people or other gender diverse options, we risk conflating cisgender and transgender people into one category and possibly misrepresenting the experiences of racial discrimination of Black gender-diverse people. This study is limited by the use of non-probabilistic sampling, which restricts the generalizability of the findings to the broader population. Additionally, the underrepresentation of men (24.4%) in the sample introduces a gender bias that may influence the interpretation and applicability of the results across different demographic groups. This limitation is particularly important given that Black men often report higher levels of discrimination compared to White men, which may not be fully captured in our findings. Finally, all the measures used in the questionnaire were self-reported, which relies on the individual’s perception and memory of their experiences.

### Implications, future research, and conclusions

This study has key implications for clinicians, institutions, and the public. It highlights the need for clinicians to consider the multiple facets of their clients’ identities in the care provided. Indeed, clinicians need to consider racial background, gender, sexual orientation, but also other sociodemographic aspects that can strengthen the impact of racial discrimination on the life satisfaction of Black individuals (Cénat et al., 2024c; Cénat et al., 2025a). The results showed it is essential to develop strategies and training programs to prevent intersectional discrimination related to race, gender, and sexual orientation. This study also shows that clinicians can play an essential role helping their clients find ways to build social capital and sociocultural resources that can mitigate the impact of racial discrimination experienced on life satisfaction (Cénat et al., 2024a). Future studies are needed to better understand the coping mechanisms of Black gender minorities in Canada who face multiple forms of discrimination, including in Black communities and gender-diverse communities. Future research should also explore other mechanisms that can help explain the life satisfaction of Black people, specifically larger samples of transgender or gender-diverse people, experiencing racial discrimination to promote better well-being and guide interventions. On a larger scale, this study also informs the need for institutions such as schools, health care settings, and workplace settings to address the systemic forms of discrimination (e.g., racism, sexism, transphobia), and barriers that facilitate everyday racial discrimination against Black people.

## Author’s note

All authors confirm that this manuscript provides a transparent report of the research. This study is related to a previous publication by the same authors titled, “*Prevalence and Effects of Daily and Major Experiences of Racial Discrimination and Microaggressions among Black Individuals in Canada*.” The previous article examined the prevalence of microaggressions, everyday racial discrimination, and major racial discrimination, as well as their combined effects on self-esteem and life satisfaction. The current study focuses specifically on the investigation of life satisfaction scores according to the sociodemographic characteristics of the sample and the association between everyday racial discrimination and life satisfaction, while exploring the mediating role of social support and the moderating roles of gender and place of birth. Both studies use data drawn from the Black Communities Mental Health in Canada Survey (BCoMHeal). The present manuscript, however, provide totally different analyses and findings by examining life satisfaction scores according to sociodemographic factors and by integrating intersectional and psychosocial dimensions to better understand the mechanisms underlying the association between everyday racial discrimination and life satisfaction.

## Data Availability

The raw data supporting the conclusions of this article will be made available by the authors, without undue reservation.
